# Reinforcing outpatient medical student learning using brief computer tutorials: the Patient-Teacher-Tutorial sequence

**DOI:** 10.1186/1472-6920-12-70

**Published:** 2012-08-08

**Authors:** Martin V Pusic, Wendy A MacDonald, Harley O Eisman, John B Black

**Affiliations:** 1 Division of Pediatric Emergency Medicine, Columbia University, 622 W 168th St, PH1-137, New York, NY 10032, USA; 2 McGill University, Department of Pediatrics, McGill University, Montreal, QC, Canada; 3 Teachers College, Columbia University, New York, NY, USA

## Abstract

**Background:**

At present, what students read after an outpatient encounter is largely left up to them. Our objective was to evaluate the education efficacy of a clinical education model in which the student moves through a sequence that includes immediately reinforcing their learning using a specifically designed computer tutorial.

**Methods:**

Prior to a 14-day Pediatric Emergency rotation, medical students completed pre-tests for two common pediatric topics: Oral Rehydration Solutions (ORS) and Fever Without Source (FWS). After encountering a patient with either FWS or a patient needing ORS, the student logged into a computer that randomly assigned them to either a) completing a relevant computer tutorial (e.g. FWS patient + FWS tutorial = “in sequence”) or b) completing the non-relevant tutorial (e.g. FWS patient + ORS tutorial = “out of sequence”). At the end of their rotation, they were tested again on both topics. Our main outcome was post-test scores on a given tutorial topic, contrasted by whether done in- or out-of-sequence.

**Results:**

Ninety-two students completed the study protocol with 41 in the ‘in sequence’ group. Pre-test scores did not differ significantly. Overall, doing a computer tutorial *in sequence* resulted in significantly greater post-test scores (z-score 1.1 (SD 0.70) in sequence vs. 0.52 (1.1) out-of-sequence; 95% CI for difference +0.16, +0.93). Students spent longer on the tutorials when they were done *in sequence* (12.1 min (SD 7.3) vs. 10.5 (6.5)) though the difference was not statistically significant (95% CI diff: -1.2 min, +4.5).

**Conclusions:**

Outpatient learning frameworks could be structured to take best advantage of the heightened learning potential created by patient encounters. We propose the Patient-Teacher-Tutorial sequence as a framework for organizing learning in outpatient clinical settings.

## Background

In existing teaching and learning models for outpatient settings, such as the One-Minute Preceptor and SNAPPS, the emphasis is placed on the preceptor-student interaction and how to extract maximal learning from this time-pressured exchange [1,2]. The One-Minute Preceptor is a teaching method triggered by the event of a learner assessing a patient. Five teaching behaviors are done by the preceptor with the goal of eliciting and modeling ideal clinical reasoning [1]. The SNAPPS model structures the preceptor-student interaction along similar lines but from more of a learner perspective [2]. In these models, outpatient learning occurs mainly through an apprenticeship model where learners are guided by experts [3,4].

**Figure 1:**
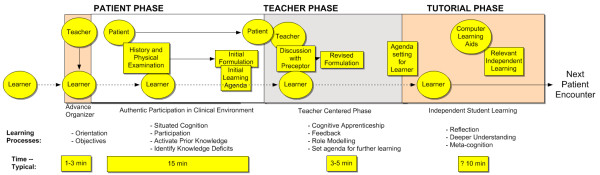
Conceptual Model for Patient-Teacher-Tutorial Sequence.

Building on these ideas, we propose the Patient-Teacher-Tutorial model of outpatient medical student learning (Figure 1) where there are three sequential phases: the medical student’s encounter with a *Patient*; their review of the patient case with the *Teacher* and, the specific subject of this paper, an independent *Tutorial* phase where the student can reinforce the learning from the patient and clinical preceptor.

### Patient encounter

Outpatient medical student learning is typically based on their clinical interviews of actual patients. The medical student-patient encounter serves to set an agenda for learning. It leads to the activation of prior knowledge about the patient’s complaint and serves to focus the attention of the learner on the content to be learned [5]. Ideally, during this phase, the student generates their own initial diagnostic and management formulation to be presented to the preceptor.

### Teacher phase

Having developed an initial formulation, the student will then discuss the patient with an expert clinician, a process that, in an Emergency Dept, typically takes less than 5 minutes with only 1-2 minutes being actual teaching of the student [6,7]. The preceptor will verify important parts of the student’s assessment and together they will arrange the necessary care for the patient [1,8]. This entire teacher-student interaction benefits from the activation of prior knowledge, identification of knowledge gaps, and high motivation. The instructional strategy is the most powerful available: individual tutoring [9]. However, it is limited by the scarce availability of the preceptor [1,6].

### Tutorial phase

Immediately after the student and preceptor have separated, the optimal conditions for learning are usually still present. Primed by both the patient encounter and the interaction with the teacher, the student is ideally placed to cement their learning. The motivated student may go on to find appropriate learning resources but in a typical clerkship there is considerable variability in the quality of the student’s search and in the resources available [10,11]. This teachable moment, which we will call the Tutorial Phase (See Figure 1), could be a shared space between the preceptor and learner, an opportunity to agree on a continuing learning agenda. This is explicitly stated in the SNAPPS model where the last “S” stands for “Select a patient-related issue for further study” [2]. However, exactly how this is to be brought about is not specified in existing learning frameworks [1,2].

In this research, we sought to study strategies for the patient-triggered use of learning resources by medical students in the clinical setting. Specifically, we wondered whether completing a brief computer tutorial right after the patient encounter can increase their learning to a measurable degree compared with completing the same tutorial but outside the Patient-Teacher sequence.

## Methods

**Figure 2:**
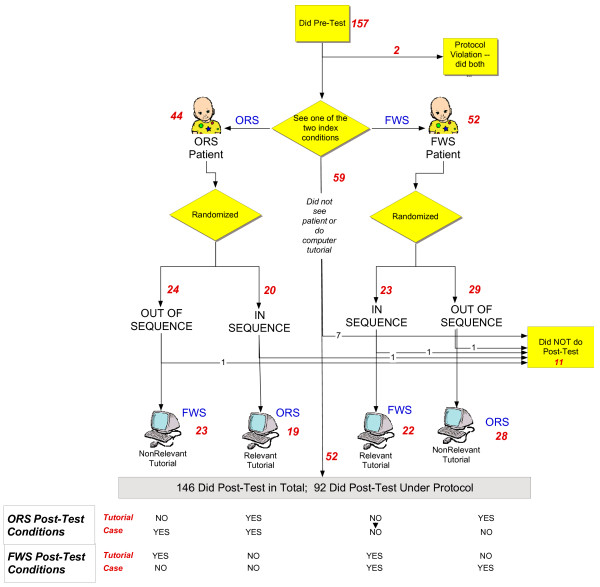
**Comparison of ‘In Sequence’ vs. ‘Out of Sequence’ Study Groups.** Depending on which index patient a participant saw, they may have ended up in any one of four combinations of having seen a patient and/or done a tutorial.

This was a randomized trial where we compared declarative knowledge examination scores for students who had seen a patient and were then randomized to doing a computer tutorial afterwards that was either relevant or not relevant to the patient seen. Our null hypothesis was that doing a relevant tutorial around the time of seeing a patient was not any more effective than doing the tutorial under otherwise similar conditions, but without the link to a specific relevant patient. The overall study design is shown in Figure 2. The McGill University IRB approved the study and written informed consent was obtained from all participants.

### Participants

All McGill University third year medical students presenting for their 2-week Pediatric Emergency Department (PED) rotation at the Montreal Children’s Hospital were eligible. There were no specific exclusion criteria.

### Computer tutorials

We designed two computer tutorials to be used by medical students in the PED. Both tutorials present mainly declarative knowledge (facts and concepts). The topics were: Oral Rehydration Solutions (ORS; objective: to describe the physiologic principles of osmosis and facilitated diffusion as they apply to oral rehydration of children – mainly conceptual knowledge) and Fever Without Source (FWS; objective: to enumerate the diagnostic and treatment approach to well-appearing febrile children aged birth-36 months – mainly factual knowledge). The design of these tutorials is reported in detail elsewhere and screen captures are supplied in Additional file 1[12]. They are brief (30 screens ORS and 42 screens FWS), potentially relevant to a patient just seen, and self-contained. The instructional strategies used included animations, schematic diagrams, interactive exercises and patient presentations but not video. We used the software authoring language Toolbook II Instructor Versions 6.5 – 8.5 (SumTotal Corporation, Mountain View, CA). The tutorials were independently reviewed by content experts and pilot tested to saturation on medical students and residents. They were installed on a single computer in the central area of the nursing station in the Pediatric Emergency Department (PED) and were not available to the students outside the study.

### Study procedure

On the first day of their rotation, the students completed the Pre-test (see Outcome Measures below). They started the study procedure when they encountered a patient in the PED with one of the two index conditions for which there were computer tutorials: any patient with vomiting and/or diarrhea ( = ORS Patient ); or any patient under 36 months of age whose fever had no readily demonstrable cause (=Fever Without Source (FWS) Patient ). After discussing the patient’s case with the preceptor and discharging the patient, but before going to see the next patient, they logged into the study computer and were randomized to one of the two study conditions. On the 12^th^-14th day of the rotation, all participants completed the two post-test examinations. Each participant completed only one of the two tutorials. Subsequent patients with FWS or ORS were managed by the students according to regular practices.

### Study maneuver

Students randomized to the *in sequence* condition did a relevant computer tutorial just after having seen the index patient (See Figure 2). For example, if they had seen a patient with fever, they would do the “Fever Without Source” computer tutorial immediately after having completed their patient write-up and discussion with their preceptor. Students randomized to the *out of sequence* study condition did the opposite computer tutorial that was not relevant to the index patient. For example, if they had seen a patient with fever, they would do the “Oral Rehydration Solutions” tutorial. The timing of presentation of the tutorial in the out of sequence group was allowed to be at a convenient point before the end of their shift and not necessarily tied to when they saw the index patient since we did not know, *a priori*, which would be best. During the rotation, study participants were only exposed to one of the two tutorials. Which tutorial an individual subject saw was dependent on the diagnosis of the index patient and on the randomized allocation to study condition. (Figure 2).

#### Allocation and randomization

Allocation to study condition was concealed from the participants until they began the tutorial. Randomization was carried out according to the procedure outlined in Figure 2. Specifically, on the first screen of the tutorial the student indicated the type of patient seen (ORS or FWS). Based on the type of patient, the student was randomized to one of the two study conditions. Randomization was by varying blocks of 6, 8, or 10. Compliance with randomization was confirmed three ways: by comparing the tutorial topics to which an individual student had been randomized to whether they had reported doing the tutorial on the exit survey; by verifying that they had a log file showing completion of the tutorial; by having the student confirm via an on-screen question that they had done the tutorial under the assigned condition for the experiment.

#### Tutorial tracking

We were able to track all interactions by the students with the computer, including the time and duration of the interaction and the titles of the screens accessed. Tutorial interactions where the student spent fewer than 4 minutes or viewed fewer than five screens were not considered to have completed the study tutorial. Date stamps on the logs allowed us to measure the number of weeks of clerkship experience.

Using the log files, we calculated the length of time that the subject spent interacting with the computer tutorial in minutes. In the PED, interruptions of some of the student sessions were inevitable. Pauses of greater than four minutes were not counted in the estimates of time spent by the students on the tutorials.

#### Outcome measures

The two tests were developed by the investigators. The topics of the questions were the two domains of Pediatric Emergency Medicine relevant to the index patient diagnoses and covered by the tutorials: ORS (6 questions) and FWS (10 questions). The same examinations were used for pre- and post-testing. The questions were a mix of multiple-choice and constructed response formats testing application of declarative knowledge (see Additional file 2). We pilot tested all the questions on 10 medical students, 12 residents, and 3 faculty pediatricians and modified them in consequence. All participants did both examinations time-limited to 15 min. After the study was completed, the examinations were de-identified and marked by a single pediatric emergency physician blind to the pre- or post-test status of the participant and blind to study group allocation.

We calculated the reliability coefficient (Cronbach’s α) for each sub-examination (ORS, FWS) based on the individuals whose data was used in the analysis of the main outcome variable [13]. We dropped 2 questions which would otherwise have impaired overall internal consistency.

#### Data analysis

In this experiment there were three independent variables with two levels each: tutorial exposure (yes, no), patient exposure (yes, no) and tutorial topic (FWS, ORS). In addition there was one continuous covariate, pre-test score, and one continuous dependent variable: post-test score. We converted the raw scores to z-scores to enable comparisons between the ORS and FWS topics [14]. We compared knowledge gain for students using a three-way (2x2x2) Analysis of Covariance where pre-test score was the covariate. If the ‘in-sequence’ tutorial completion was superior to ‘out-of-sequence’ completion, then we anticipated seeing a statistically significant Patient Exposure by Tutorial interaction. We report the results in terms of means of standardized scores (percentages or z-scores), as well as using effect sizes (Cohen’s *d*) with 95 % confidence intervals based on the non-central t distribution as calculated using the ESCI software designed by Cummings and Finch [15].

Students who did not complete the protocol including post-test could not be analyzed on the main dependent variable. For non-completers, we tracked their pre-test scores and their rate of completion of the tutorials for comparison with the group that did complete the post-test.

#### Sample size

We had pilot information from a previous trial using similar short-answer questions [12] and so calculated a sample size based on potential post-test score differences between groups. We chose a minimally important effect size of 0.4 and a sample size of 50 per group as being the best balance of resource use and protection against Type II error, assuming β=0.8 and α=0.05.

## Results

### Characteristics of study population

The study flow diagram is shown in Figure 2. Between Aug 2008 and June 2009, 157 students did the Pre-Test of whom 96 (61%) saw a patient with one of the two index diseases and logged into the study computer to be randomized. Participants did not differ from non-participants in terms of Sex or Pre-test score.

The randomization resulted in 43 students being assigned to the ‘in sequence’ condition while 53 were in the ‘out of sequence’ condition. Four students did not complete the post-test so that we had 92 students complete the entire study maneuver with 51 in the ‘out of sequence’ condition and 41 ‘in sequence’. These two groups did not differ significantly in terms of Sex, Clerkship Experience or Pre-test Scores.

### Outcome instrument

One question was dropped from each of the ORS & FWS tests due to poor point-biserial correlations. The reliability of the knowledge test as measured on subjects who completed the entire study maneuver (N = 92) was 0.84 (Cronbach’s alpha) for the ORS subscale (5 questions) and 0.63 for the FWS subscale (9 questions).

### Baseline measures

There were no differences in baseline measures for the randomization groups (in-sequence vs. out-of-sequence) in terms of sex (60% female vs. 57%), and pre-test scores (30% vs. 32%).

Students who were able to complete a tutorial scored higher on the post-test (54.0% (SD 13%) vs. 37.0% (12.5%) than did the 52 who did the post-test but not a tutorial (95% CI for difference +12.5%, +21.5%).

Across all study groups, the 144 participants who completed both tests had an average combined raw score that increased from pre-test 31% (SD = 11%) to post-test 48% (SD = 15.5%). This difference corresponds to a Cohen’s d of 1.1 indicating a large effect size and is statistically significant (p ≪ 0.001).

### Effect of time since patient encounter

For the ‘out of sequence’ subjects, the timing of when they did the tutorial was variable with 26 of 51 (49%) doing the non-relevant tutorial immediately after seeing the index patient while the rest did the tutorial some convenient time later in their 8-hour shift. This timing difference did not affect post-test scores to an educationally or statistically significant degree (Post-test Raw Scores: ORS: Immediate 61% vs. Later in Shift 63%; FWS 56% vs. 55%). All subsequent analyses do not distinguish between ‘out of sequence’ subjects on the basis of when they completed the tutorial during the shift.

### Knowledge gain by study condition

**Figure 3:**
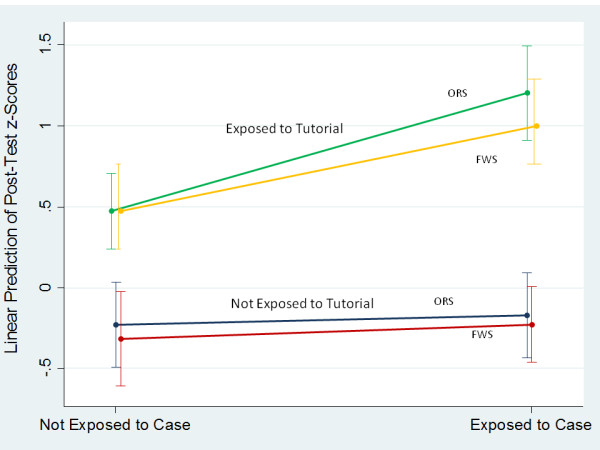
**Margin Plot Showing Interaction Between Tutorial and Patient Exposure Status.** Margins plot for the main ANCOVA model shown in Table 2. Points are means with 95% confidence intervals indicated by the error bars. Tutorial exposure raised Post-Test scores in all groups but especially for those who had been exposed to a Patient. ORS: Oral Rehydration Solutions topic. FWS: Fever Without Source topic.

For our main outcome, we compared z-scores for all tutorial completions, irrespective of topic using an ANCOVA model as shown in Table 1. Adjusting for pre-test score, the model showed significant main effects for Tutorial, Patient Exposure and the interaction term between Patient Exposure and Tutorial suggesting that *in sequence* condition has a significant effect on post-test score over and above that independently predicted by seeing a relevant patient or doing the tutorial in isolation. (See Table 1, Figure 3).

**Table 1:** ANCOVA Table showing the interplay between Patient Exposure (Yes, No), Tutorial Completion (Yes, No) and Topic (Oral Rehydration Solution = 0, Fever Without Source = 1) all adjusted for Pre-Test Score

**Source**	**Partial Sum of Squares**	**df**	**Mean Square**	**F**	**Significance**
Model	62.96	8	7.87	11.40	0.00
Pre-test z-Score	12.33	1	12.34	17.87	0.00
Patient Exposure	6.22	1	6.22	9.01	0.00
Tutorial Completion	42.34	1	42.34	61.34	0.00
Topic	.45	1	.45	0.65	0.42
Patient * Tutorial	3.34	1	3.34	4.84	0.03
Patient * Topic	.11	1	.11	0.16	0.69
Tutorial * Topic	.00	1	.00	0.01	0.93
Patient * Tutorial * Topic	.19	1	.19	0.28	0.60
Error	120.8	175	0.69		
Total	183.8	183	1.00		

Consistent with the study hypothesis, there is a statistically significant interaction between Patient Exposure and Tutorial Completion.

**Table 2:** Post-test z-scores by study group

**Topic**	**Tutorial + Relevant Patient +**			**Tutorial + Relevant Patient –**			**Tutorial – Relevant Patient +**			**Tutorial – Relevant Patient –**			**Statistical Significance**
	**N**	**M**	**SD**	**N**	**M**	**SD**	**N**	**M**	**SD**	**N**	**M**	**SD**	
ORS Post-test	19	1.23	0.51	28	0.51	1.0	23	-0.18	0.71	22	-0.17	0.93	*F* = 4.0 (1,87); p ≪ 0.05
FWS Post-test	22	0.91	0.81	23	0.53	1.9	28	-0.15	0.77	19	0.33	0.82	*F* = 1.7 (1,87); p = NS
OVERALL	41	1.06	0.70	51	0.52	1.1	41	-0.24	0.88	51	-0.17	0.74	*F* = 4.8 (1,175); p = 0.03

· ORS = Oral Rehydration Solutions topic; FWS = Fever Without Source topic.

· N = number of participants; M = mean z-score; SD = Standard Deviation.

· For the ORS and FWS Post-test scores, the F-tests are for the interaction of Case with Tutorial for the partial ANCOVA model for the respective topic while the Overall F-test is from Table 1.

· All analyses adjusted for pre-test score.

· ORS cells are yoked to FWS cells (and vice-versa) per the study design (see Figure 2); for example, all students who were Tutorial +, Patient + for the FWS .Post-test were Tutorial – and Patient – for the ORS Post-test.

None of the terms that included Topic (ORS vs. FWS) were statistically significant suggesting that knowledge gain was not moderated by which of the two topics is considered.

When the results are broken out by topic, the same pattern is observed as in the overall model, with increased test scores being associated with the *in sequence* condition. This effect reached statistical significance for the ORS topic where students who did the tutorial in the *in sequence* condition scored 19% higher (95% confidence interval for the difference +7%, +31%) corresponding to a large Cohen's-d effect size of 0.85 (95% CI 0.25, 1.5). Students who did the FWS tutorial in the *in sequence* condition scored an average of 6% HIGHER on the post-test than the comparison group (95% confidence interval for the difference (-3%, +16%) corresponding to a Cohen's-d effect size of 0.38 (95% CI -0.20, +0.97) which is not statistically significant.

### Comparison of time on tutorial by study condition

As a planned subgroup analysis, we examined whether the time spent interacting with the tutorial varied according to whether it was done ‘in sequence’. We found estimating and interpreting the actual amount of time spent by the student from the log files difficult. In more than 25% of the cases, there appeared to be long periods between student clicks on the screens. We did not include these periods in our time estimates if they extended beyond four minutes. Our final estimate found that, when adjusted for which tutorial was being done, the *‘in sequence’* group spent 1.6 minutes more with a given tutorial than did the comparison group (95% Confidence Interval -1.2, + 4.5 minutes), which is not statistically significant.

## Discussion

We have proposed a framework for learning in clinical settings which extends current models. We found that presenting a computer tutorial immediately after a patient-preceptor encounter lead to measurably greater learning compared with doing *the same tutorial* separately from seeing a patient. This confirms the conventional wisdom of reading around cases but also suggests that clinical educators can purposefully design a Patient-Teacher-Tutorial sequence to take advantage of this maxim.

Current models of outpatient teaching and learning describe optimal teacher and learner behaviors that can increase the amount of learning during a given patient encounter. The two most widely studied models, the One-Minute Preceptor and the SNAPPS models, do not specifically describe what is to be done by the student after separation from the preceptor at the end of the patient interaction [2,8,16]. Harden et al. at the University of Dundee have done considerable work on the integration of computer-based learning into the clinical rotations of medical students [17]. They enumerated over 130 tasks which could be done by students over the course of their clinical rotations. The tasks are supported with a series of customized study guides that highlight important objectives and specify available learning resources that the student may use in their learning about the task [17]. However, the nature of the interaction between the study guides and the students’ patient experience is not reported in detail.

Our suggested Patient-Teacher-Tutorial (PTT) Sequence, composed of *patientteacher* and *tutorial* phases, integrates with and extends these existing models. The PTT model takes into account the constraints imposed by the workplace setting on the learning of conceptual knowledge. Given the time-limited and goal-oriented nature of their interaction, the clinician and student will tend to focus on content that gets the patient encounter done [18]. While this is as it should be, Billett and others have pointed out that, for a student to function completely in the workplace, they need access to the “opaque or hidden” conceptual knowledge that is necessary for a flexible knowledge base [19]. Consider the example of a patient with dehydration due to diarrhea. The student sees the patient and reports back to the preceptor, saying that the patient needs hydration with oral fluids. In the ensuing discussion, the clinician would focus on the choice of the fluid (an Oral Rehydration Solution) and how the patient should take it (e.g. one teaspoon at a time, spaced at 5 min intervals etc…). This directive information will ensure that the patient is correctly instructed. However, what is hidden are the conceptual underpinnings for this directive advice. Oral rehydration solutions are constructed according to a complex understanding of physiologic processes at the level of the intestinal absorptive cells and how they function when damaged by a virus. Understanding these physiology concepts will allow the future clinician to make finer grained judgments as to which fluids to use in which situations.

If we accept that students need to learn both the procedure-specific knowledge and the conceptual underpinnings to optimally function in the clinical setting, then the issue becomes one of ensuring that both types of learning occur. One option might be to simply expand the role of the preceptor to ensure that they include discussions of the conceptual as well as the procedural. However, preceptor time considerations often preclude this more symbolic, conceptual knowledge teaching [6,7,18] but, with support, the student can learn this material on their own from well-designed learning aids [12,20-24].

An attractive aspect of the PTT sequence is that the teacher facilitates the student’s relationship with the declarative knowledge without necessarily being the provider of it. This continually reinforces the way of being of an expert practitioner [25]. Expert practitioners require ready access to domain specific declarative knowledge [26]. Ely estimates that practicing physicians have one clinically important knowledge question for every 3 patients they see [26,27]. The diligent pursuit of these knowledge needs is a cornerstone of both evidence-based practice and of lifelong learning [28,29]. Assiduously mandating that medical students do in-stream, focused reading after each patient could potentially establish a lifelong adaptive pattern.

The following limitations should be borne in mind while considering this study. It required the voluntary participation of busy medical students on a difficult rotation. A significant minority did not participate leaving the results susceptible to volunteer bias. We have presented a *per protocol* analysis. While we analyzed what we randomized, a sizeable minority of the students did not participate. However, this new unconventional technique might not suffer from the same non-completion rate once it is established that it can work and becomes part of the regular workflow, instead of being presented as a supplemental research protocol. We likely had sizeable variability between faculty preceptors in terms of their teaching on these topics. This variability could impact the intervention in unknown ways. Some of our comparisons were under-powered, especially the FWS subanalysis where the multidimensionality of the test would have decreased the power of the comparison.

RCTs in educational settings are controversial with some holding them up as the gold standard [30] while others deride them as hopelessly confounded [31]. We chose this design in order to tightly contrast the study interventions based on a pre-specified conceptual model.

Our tutorials were invariant “black boxes” in these studies. As such, they used fixed instructional strategies that may not necessarily have been optimal for a given student situation. There are ways in which a computer tutorial can adapt to a student’s particular prior knowledge or personal educational goals [32]. Such adaptations might have made the tutorials more effective and might have interacted with our study manipulations in important ways. We did not have a measure of knowledge retention beyond the rotation.

There may be unforeseen disadvantages to the intervention itself. We postulate that it is a third-best educational option behind learning from patients and learning from preceptors. If the intervention results in the learner seeing fewer patients or spending less time with their preceptors, this would be deleterious. By geographically stationing the computer tutorials right in the nursing station and by temporally presenting them right in the flow of patients, we believe that they can be done with a minimum of disruption to the learner’s interactions with their mentors and patients [12,17,33].

## Conclusions

We have presented empiric data to support a framework for medical student outpatient learning that extends current models. A computer tutorial done in conjunction with seeing a relevant patient (i.e. the Patient-Teacher-Tutorial sequence) is more effective in raising test scores than the same tutorial done separate from a patient encounter. It could be educationally profitable to restructure outpatient learning frameworks to complement limited preceptor time with focused computer tutorials.

This work was presented as an abstract at the Association of American Medical Colleges Annual Meeting, November 2010, Washington DC.

## Competing interests

The authors declare that they have no competing interests.

## Authors’ contributions

MVP conceived of the studies wrote the computer tutorials, carried out the analyses and drafted the manuscript. WAM and HE participated in study design, carried out the study, and reviewed the data and the entire manuscript. JBB participated in the design and analysis of the study, as well as reviewing the manuscript. All authors read and approved the final manuscript.

## Pre-publication history

The pre-publication history for this paper can be accessed here:

http://www.biomedcentral.com/1472-6920/12/70/prepub

## Supplementary Material

Additional file 1 **Tutorial Screen Captures.** Screen captures from both computer tutorials used in the study. Click here for file

Additional file 2 **Test questions.** All test questions used in the study. Click here for file
