# Supplementary material for: Reinforcing outpatient medical student learning using brief computer tutorials: the Patient-Teacher-Tutorial sequence
Source: BMC Med Educ. 2012 Aug 8;12:70. doi: 10.1186/1472-6920-12-70 (PMC3517358; doi:10.1186/1472-6920-12-70)
Supplement: Additional file 1 — Tutorial Screen Captures. Screen captures from both computer tutorials used in the study. [file 1472-6920-12-70-S1.pdf]

The Just-in-Time Learning Study

The objective of this study is to examine the ways computer tutorials can support learning in the Emergency Dept. You will be randomly assigned one of two computer tutorials to be done either now, or in a delayed fashion.

☐ I certify that I am a McGill medical student who has signed written consent to participate in the Just-in-Time Learning Study.

Start

If you are not a McGill medical student please Close Application...

Close Application

The Just-in-Time Learning Study

Please enter the following information and then click the "Login" button

LAST NAME
Your name will not be logged

FIRST INITIAL:

CODE WORD:
The one used on the test

Login

If you are not a McGill medical student please Close Application...

Close Application

Introduction

Fever in young children who otherwise look well (i.e. are non-toxic) is a common clinical dilemma.

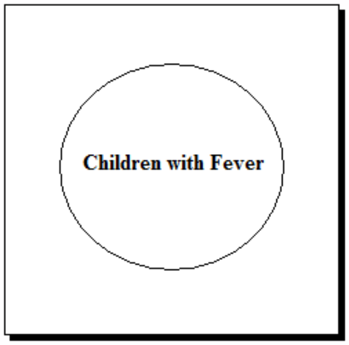

Introduction

In the majority of cases, the cause is viral.

The rate of complication in viral cases is low.

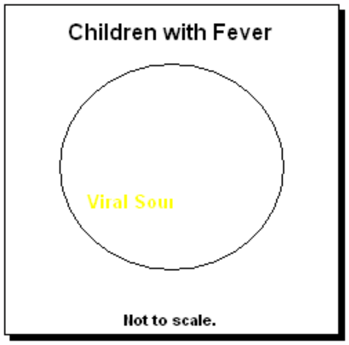

Introduction

However, a certain number of these cases turn out to be due to bacterial causes.

The complication rate in this group is significant.

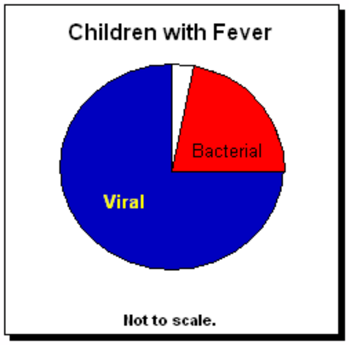

Introduction

For the usual acute individual case, a clinician attempts to classify the etiology as either viral or bacterial.

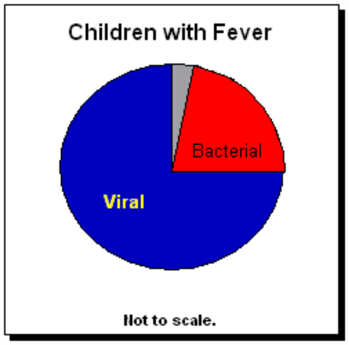

Introduction

Viral causes have a low complication rate and usually need only supportive therapy.

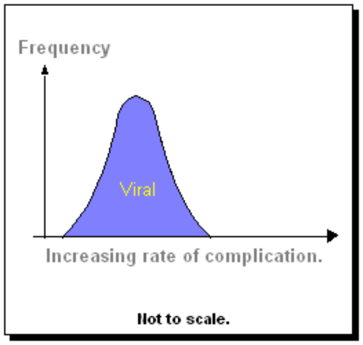

Introduction

Bacterial disease, with its higher complication rate, is managed more aggressively.

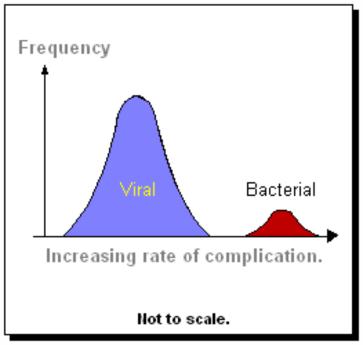

Introduction

The basic idea is to use all of the clinical information available to us to assign a given patient to one of these two categories and then to treat accordingly.

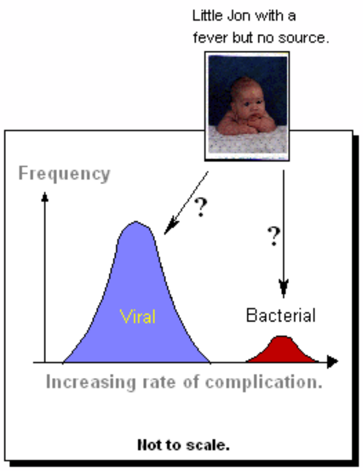

Introduction

Unfortunately, real life does not always lend itself to such neat divisions.

There exists a group of children for whom no distinction can be made. For these children, the cause of the fever, viral or bacterial, is not clear.

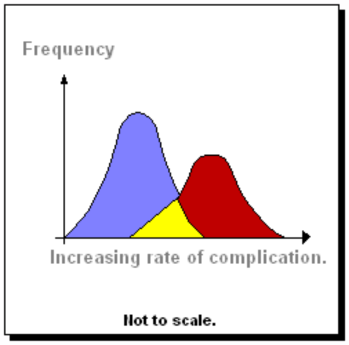

Introduction

This overlapping group, children who have "Fever Without Source", has been extensively studied.

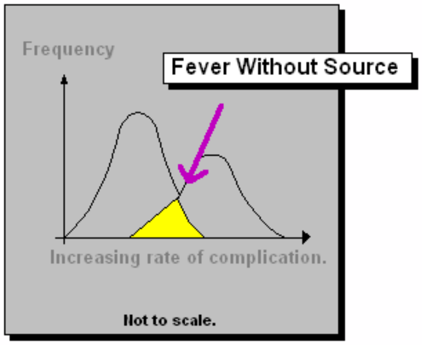

Introduction

This overlapping group, children who have "Fever Without Source", has been extensively studied.

However, to date, no combination of history, physical and laboratory parameters discriminates between benign and malignant causes of fever with adequate sensitivity and specificity.

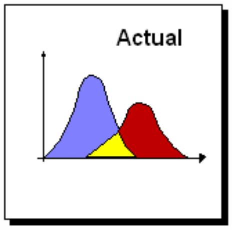

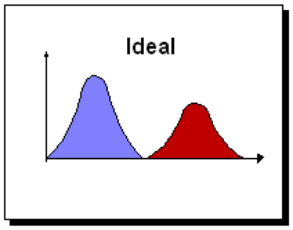

Introduction

Because of our inability to classify children who have Fever Without Source, some of these children have been under- or over-treated.

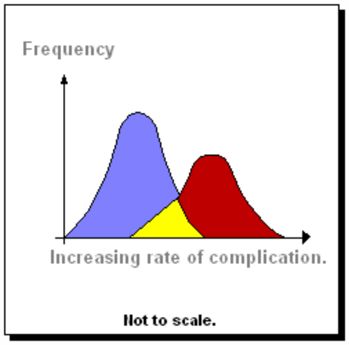

Objectives

At the end of this tutorial the student will be able to:

Discuss the difference in approach to FWS depending on age.

Suggest which investigations are warranted in FWS.

Discuss the probabilities of serious sequelae from FWS.

Treatment of FWS in Infants < 28 Days

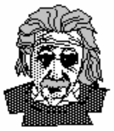

Because we can not tell based on clinical grounds which child will have complications and which child will not, we have to consider them as a population and not as individuals.

It does help to subdivide this population of children based on their age.

In general, the younger the child, the greater the risk of complications.

A One-Month Old

A Two-Month Old

A Three-Month Old

A Three-Year Old

Summary

Let's consider some cases...

- A One-Month Old
- A Two-Month Old
- A Three-Month Old
- A Three-Year Old
- Summary

A One-Month Old

A 25-day old infant presents to you having had a fever during the day. Apart from mild rhinitis, the child has otherwise been asymptomatic. The rest of the family all have cold symptoms. Physical examination reveals a perfectly-well child save for a Temperature of 38.2C taken rectally.

Which of the following would you do?

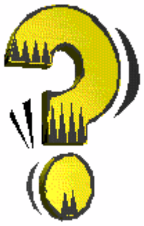

- Full Septic Work-up and Admit Automatically
- Full Septic Work-up and Admit if Positive
- Partial Septic Work-up - Further workup if abnormal
- No workup necessary

-- Left-click over the diamond which corresponds to your answer --

Treatment of FWS in Infants < 28 Days

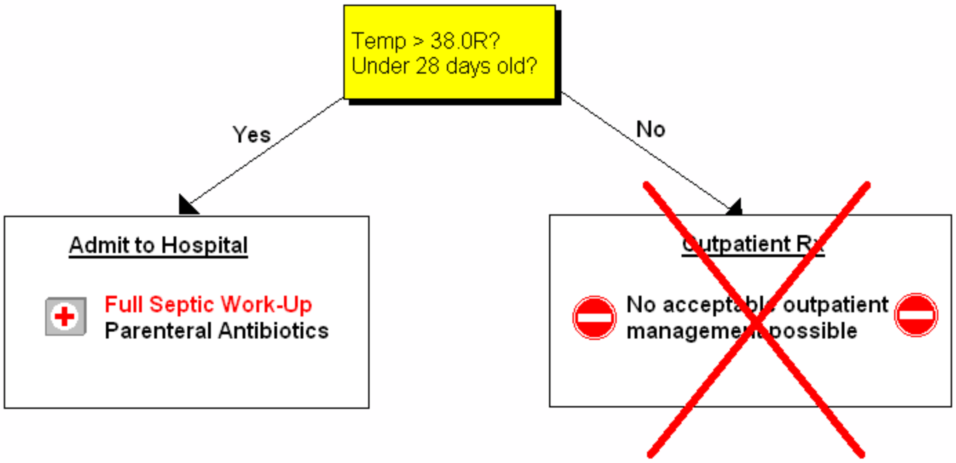

Treatment of FWS in Infants < 28 Days

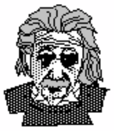

Children under 28 days are a special group.

Essentially, regardless of other clinical information, their age and the fact they have a temperature earns them a full septic work-up and admission to hospital for i.v. antibiotics.

The rationale for this is that these children can become moribund within a few hours especially with Group B Strep and E. coli sepsis.

One - Three Months

<u>One-Three months</u>

The infants aged 1-3 months are still at high risk but less so than neonates are.

Consider this case...

A One-Month Old

A Two-Month Old

A Three-Month Old

A Three-Year Old

Summary

A Two-Month Old

A 6-week old infant presents to you with the same scenario as the previous child: mild rhinitis; otherwise asymptomatic; the rest of the family all have cold symptoms. Physical examination is normal except a temperature of 38.2C taken rectally.

Which of the following would you do?

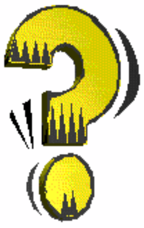

- **Full Septic Work-up and Admit Automatically**
- **Full Septic Work-up and Admit if Positive**
- **Partial Septic Work-up - Further workup if abnormal**
- **No workup necessary**

-- Left-click over the diamond which corresponds to your answer --

Treatment of FWS in Infants 28 - 90 Days

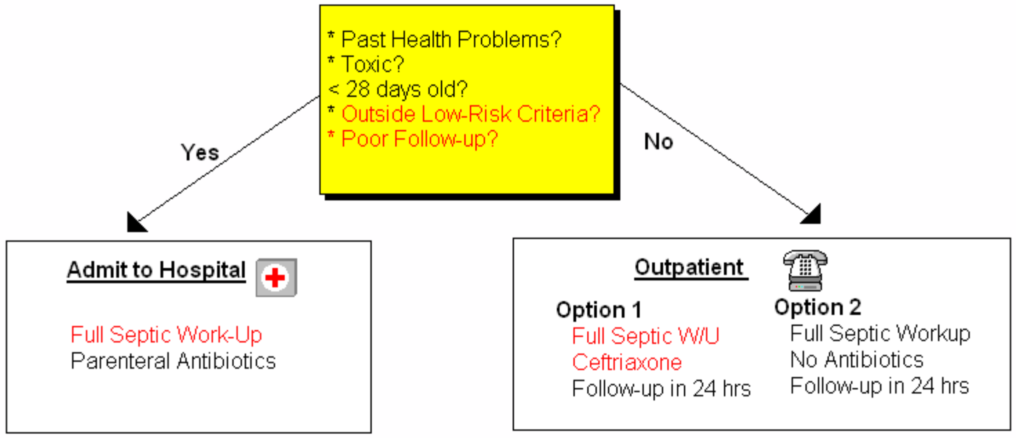

Click here for a note on follow-up.

FWS in Infants 28 - 90 Days

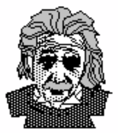

The key to children 28-90 days of age are the low-risk laboratory criteria.

They allow us to select which children can safely be discharged *assuming adequate follow-up*.

Note that the low-risk criteria require a lumbar puncture.

LOW-RISK CRITERIA:

Clinical:
- previously healthy
- nontoxic appearance
- no focal bacterial infection (except OM)

Laboratory:
- WBC 5,000 - 15,000
  < 1500 bands
- Normal CSF
- Normal urinalysis
- Diarrhea: < 5 WBC/hpf

3-36 Months

Well-appearing children 3-36 months of age can usually be managed on an outpatient basis.

Who to investigate and the role of prophylactic antibiotics is controversial.

Let's have a look...

A One-Month Old

A Two-Month Old

A Three-Month Old

A Three-Year Old

Summary

A Three-Month Old

A 3-month old child presents to you with the same scenario as the previous infant except the temperature is higher at 39.5C. The WBC is measured and found to be 16,000.

mild rhinitis; otherwise asymptomatic; the rest of the family all have cold symptoms; physical examination is normal.

What is the probability that this child will suffer permanent sequelae as a result of this illness?

- 1: 500
- 1:1000
- 1: 2000
- 1: 5000

-- Left-click over the diamond which corresponds to your answer --

3-36 Months

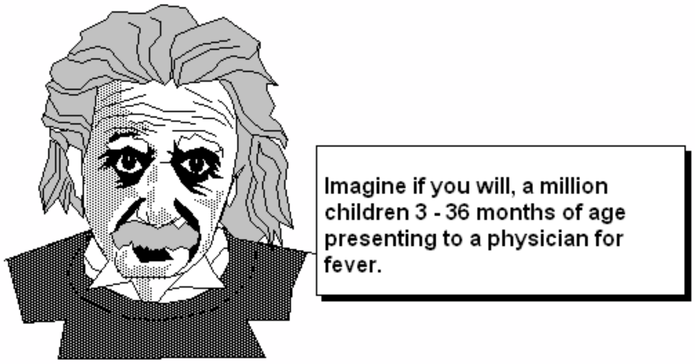

3-36 Months

1,000,000 Children with Fever.

3-36 Months

1,000,000 Children with Fever.

3-36 Months

1,000,000 Children with Fever.

To make investigation and treatment cost-effective, the authors selected a sub-population at higher risk.

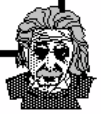

3-36 Months

1,000,000 Children with Fever.

To make investigation and treatment cost-effective, the authors selected a sub-population at higher risk.

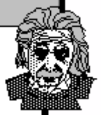

The two best are:

Height of Temperature
WBC

3-36 Months

1,000,000 Children with Fever.

What percentage of the million children will have a temperature > 39°C ?

- 10%
- 25%
- 33%
- 50%

Left-Click the diamond opposite your answer.

1,000,000 Children with Fever.

≥ 39C ?

333,333 Children with Fever > 39°C

Now, what percentage of these children (T > 39°C) will have a WBC > 15,000 ?

- 10%
- 25%
- 33%
- 50%

1,000,000 Children with Fever.

$\geq$ 39C ?

333,333 Children with Fever > 39°C

WBC $\geq$ 15,000 ?

100,000 Children with Fever > 39°C & WBC > 15k

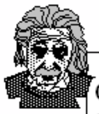

Certain expert groups feel that this sub-group should be routinely treated with antibiotics. This is the subject of considerable controversy.

Relatively few practitioners do so, relying instead on close follow-up.

1,000,000 Children with Fever.

$\geq$ 39C ?

333,333 Children with Fever > 39°C

WBC $\geq$ 15,000 ?

100,000 Children with Fever > 39°C & WBC > 15k

What percentage of the target population would prove to be bacteremic?

- 2%
- 4%
- 6%
- 8%

1,000,000 Children with Fever.

↓ ≥ 39C ?

333,333 Children with Fever > 39°C

↓ WBC ≥ 15,000 ?

100,000 Children with Fever > 39°C & WBC > 15k

↓ BACTEREMIA ?

2000 Bacteremic Children

↙ No Rx

120 Meningitis

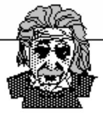

6% of these would develop pneumococcal meningitis...

A much smaller number would develop meningococcal or hemophilus meningitis but that is not considered in the analysis.

1,000,000 Children with Fever.

↓ ≥ 39C ?

333,333 Children with Fever > 39°C

↓ WBC ≥ 15,000 ?

100,000 Children with Fever > 39°C & WBC > 15k

↓ BACTEREMIA

3,000 - 11,000 Bacteremic Children

↙ No Rx

120 Meningitis

↓

50 Sequelae

... and of those who developed pneumococcal meningitis, 42% would suffer moderate or serious sequelae or death.

- click here for the beakdown of Sequelae -

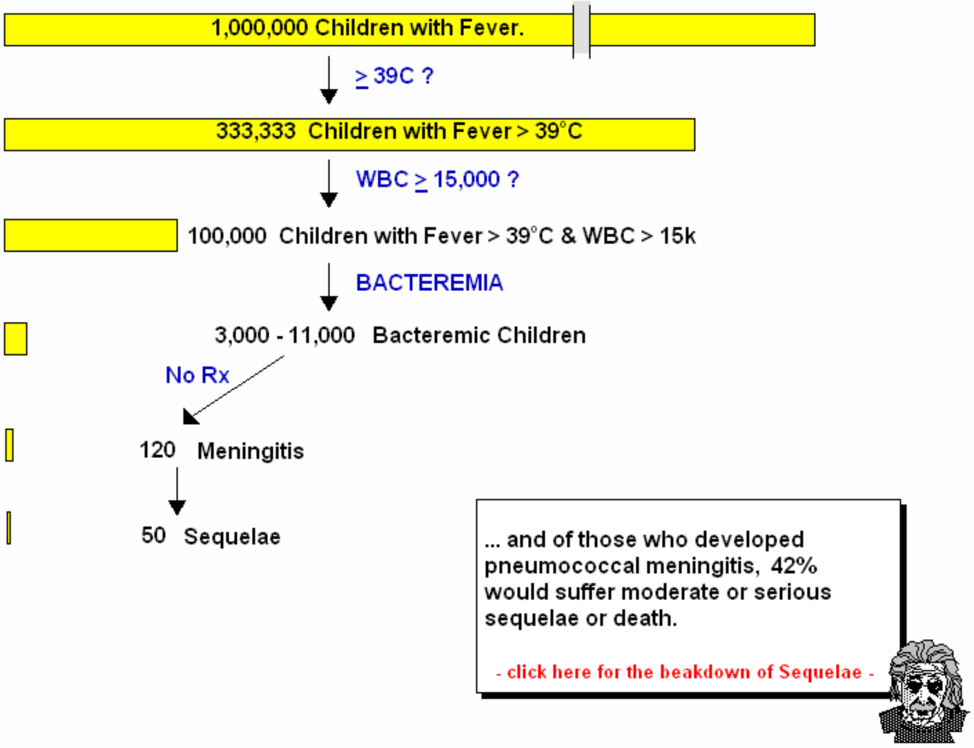

1,000,000 Children with Fever.

$\geq$ 39C ?

333,333 Children with Fever > 39°C

WBC $\geq$ 15,000 ?

100,000 Children with Fever > 39°C & WBC > 15k

BACTEREMIA ?

3,000 - 11,000 Bacteremic Children

No Rx

120 Meningitis

50 Sequelae

Put another way, at worst 1 serious sequela occurs for every 2000 patients seen with fever > 39°C and WBC > 15,000.

- click here to see the math -

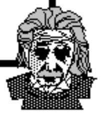

So what should we do with our patient?

A 3-month old child presents to you with the same scenario as the previous infant except the temperature is higher at 39.5C.

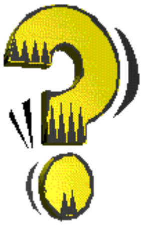

Treatment of FWS in Children 3-36 Months

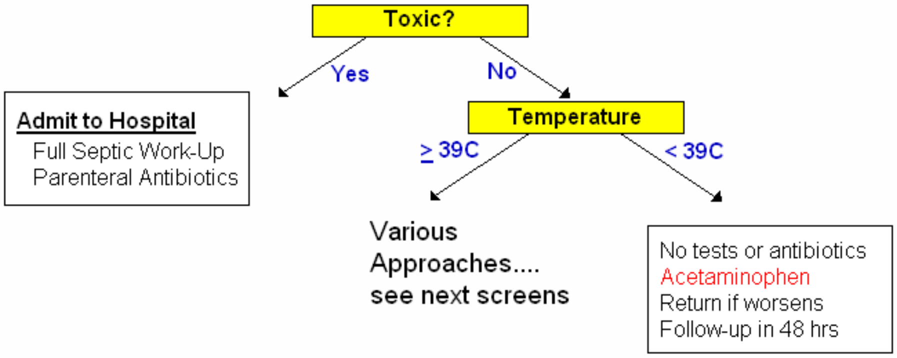

Note that this guideline is meant to apply to children who have been previously healthy and for whom follow-up is available.

So what should we do with our patient?

A 3-month old child presents to you with the same scenario as the previous infant except the temperature is higher at 39.5C.

Risk Minimizers

Some experts suggest measuring the WBC on all children 3-36 months whose T > 39.0C. If the WBC > 15,000 then a blood culture is sent and IM Ceftriaxone is given with follow-up in 24 hours.

Treatment of FWS in Children 3-36 Months

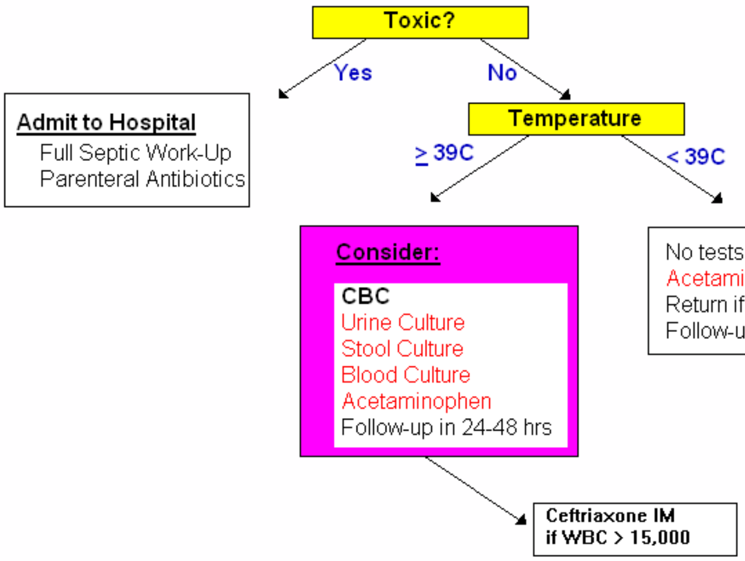

Note that this guideline is meant to apply to children who have been previously healthy and for whom follow-up is available.

So what should we do with our patient?

A 3-month old child presents to you with the same scenario as the previous infant except the temperature is higher at 39.5C.

Risk Minimizers

Some experts suggest measuring the WBC on all children 3-36 months whose T > 39.0C. If the WBC > 15,000 then a blood culture is sent and IM Ceftriaxone is given with follow-up in 24 hours.

Test Minimizers

An alternative approach is to arrange **close follow-up (q24hrs) without specific investigations or antibiotic prophylaxis.**

Treatment of FWS in Children 3-36 Months

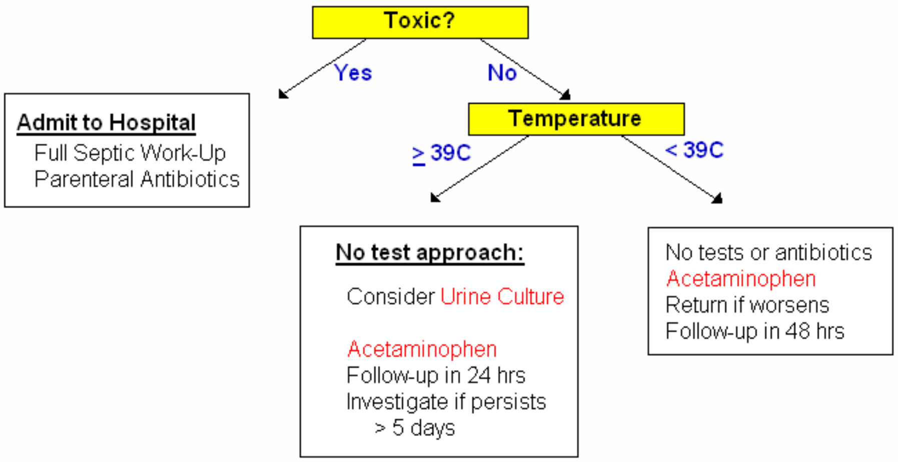

Note that this guideline is meant to apply to children who have been previously healthy and for whom follow-up is available.

So what should we do with our patient?

A 3-month old child presents to you with the same scenario as the previous infant except the temperature is higher at 39.5C.

Risk Minimizers

Some experts suggest measuring the WBC on all children 3-36 months whose T > 39.0C. If the WBC > 15,000 then a blood culture is sent and IM Ceftriaxone is given with follow-up in 24 hours.

Test Minimizers

An alternative approach is to arrange close follow-up (q24hrs) without specific investigations or antibiotic prophylaxis.

Both approaches are used at this institution. Remember to solicit the input of the parents of your patient.

So what should we do with our patient?

A 3-month old child presents to you with the same scenario as the previous infant except the temperature is higher at 39.5C.

One thing that both groups agree upon is the need to consider urinary tract infection.

Girls less than 2 years of age and boys less than 12 months run a roughly 5% risk of UTI.

A urinalysis and culture should be considered for any child with persisting fever.

Summary

<u>3-36 Months</u>

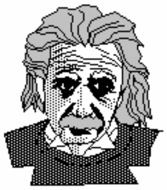

Well-appearing children 3-36 months of age can usually be managed on an outpatient basis.

Height of fever and WBC can be of moderate help in discerning children at risk. Close follow-up is probably more important to ultimate good outcome.

Summary

Remember these important points...

The End

GREAT Work!

You have successfully completed the tutorial. Please answer these final questions and then click on the pink "EXIT" button to complete the tutorial.

1

Your index case for entering the study had **FEVER UNDER 36 MONTHS.** In your opinion, how relevant was this tutorial to the case you saw?

- Not at all RELEVANT
- Minimally RELEVANT
- Somewhat RELEVANT
- Very RELEVANT
- Completely RELEVANT

2 Is there another topic/issue that you would rather have learned about?

- NO
- YES

3 If so, what would that have been?

4 SUBMIT & EXIT

The End

GREAT Work!

You have successfully completed the tutorial. Please answer these final questions and then click on the pink "EXIT" button to complete the tutorial.

1 Your index case for entering the study had **DIARRHEA AND/OR VOMITING.** In your opinion, how relevant was this tutorial to the case you saw?

- Not at all RELEVANT
- Minimally RELEVANT
- Somewhat RELEVANT
- Very RELEVANT
- Completely RELEVANT

2 Is there another topic/issue that you would rather have learned about?

- NO
- YES

3 If so, what would that have been?

4 SUBMIT & EXIT

British Columbia's Children's Hospital

UBC

Oral Rehydration Therapy

Start

Pediatric Emergency Medicine
British Columbia Children's Hospital
University of British Columbia
Vancouver, B.C.

© Copyright 2003 Martin V. Pusic
All Rights Reserved.

Oral Rehydration Therapy

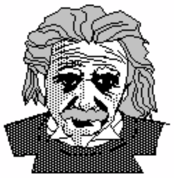

The idea of orally replacing the fluid lost in diarrhea is not new. Ancient Hindu texts describe solutions for that purpose, and virtually every culture has a heritage of "grandmother solutions", such as chicken soup or coconut juice, that contain salt, sugar, starches, and proteins.

Oral Rehydration Therapy

The objective of this brief tutorial is to explain, on a pathophysiological basis, the rationale for using Oral Rehydration Solutions (ORS).

Section 1

Introduction

Why is ORS better than other fluids for children with diarrhea?

- ORS is less osmotically active
- ORS is more nutritious
- ORS takes advantage of passive diffusion
- ORS takes advantage of facilitated transport

Introduction

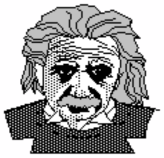

There are two main mechanisms to get fluid across the GI mucosa into the blood...

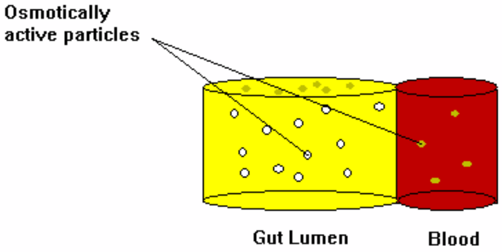

Introduction

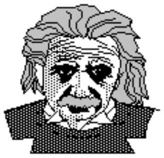

There are two main mechanisms to get fluid across the GI mucosa into the blood...

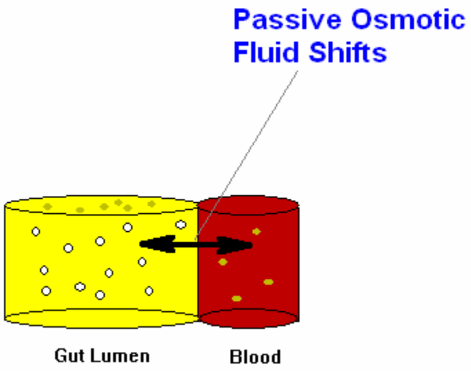

Introduction

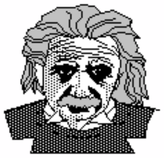

There are two main mechanisms to get fluid across the GI mucosa into the blood...

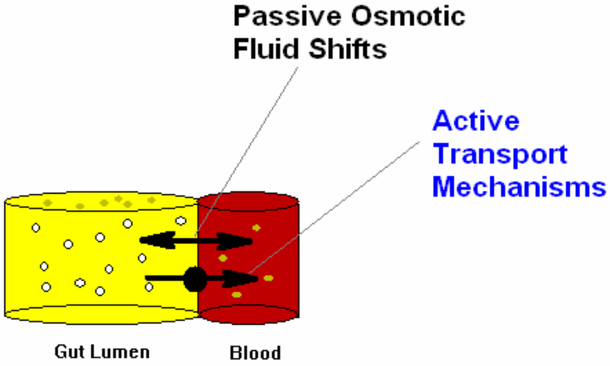

Section 2

Advantages of ORS

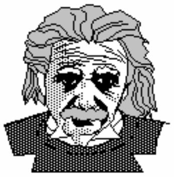

Oral rehydration solutions have 2 advantages over other available fluids:

1. ORS causes less in the way of deleterious passive osmotic fluid shifts.

Advantages of ORS

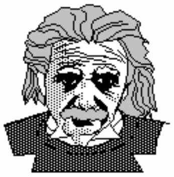

Oral rehydration solutions have 2 advantages over other available fluids:

1. ORS causes less in the way of deleterious passive osmotic fluid shifts.
2. ORS takes best advantage of the active absorptive processes that remain intact.

Advantages of ORS

Let's look more closely at these two advantages of ORS...

Section 3

Osmotic Fluid Shifts

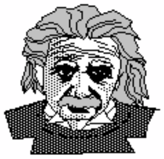

ORT minimizes deleterious passive osmotic fluid shifts.

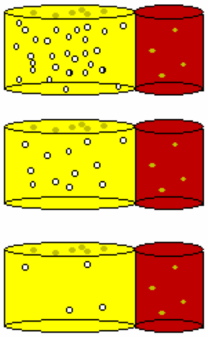

Osmotic Fluid Shifts

Consider the case where our rehydration fluid is HYPER-osmolar.

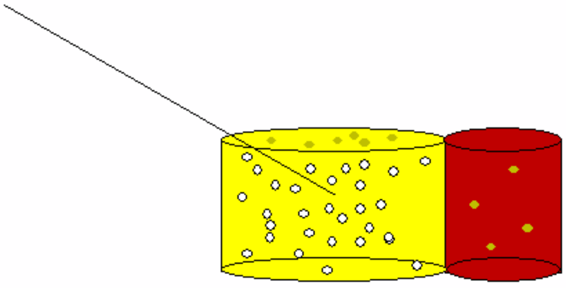

Osmotic Fluid Shifts

Consider a HYPER-osmolar ORS solution.

By dragging the appropriate arrow over to the figure, show which way water would flow in response to the osmotic gradient shown.

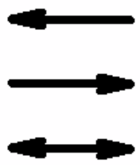

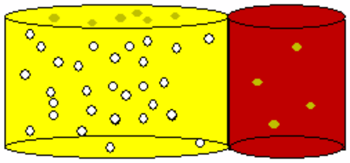

Programmed drag & drop

Reset

Osmotic Fluid Shifts

Apple Juice has an osmolarity of roughly 800mOs/L which is almost 3x that of serum.

As a result, it causes a deleterious osmotic flow from (dehydrated) blood to the lumen of the intestine.

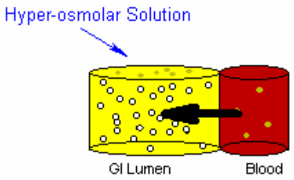

Osmotic Fluid Shifts

Next consider a HYPO-osmolar rehydration solution.

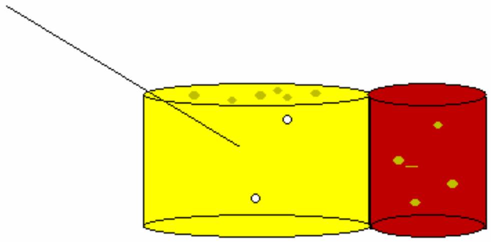

Osmotic Fluid Shifts

Consider a HYPO-osmolar ORS solution.

Show which way water would flow in response to the osmotic gradient shown, by dragging the appropriate arrow over to the figure.

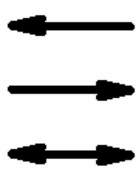

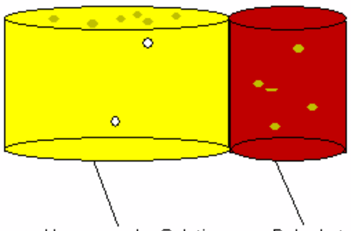

Reset

Programmed drag & drop

Osmotic Fluid Shifts

Put the following list of fluids in increasing order of osmolarity.

Drag the image into the appropriate box

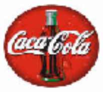

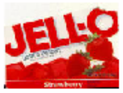

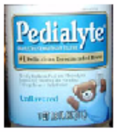

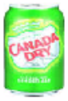

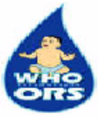

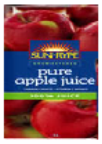

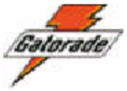

Definable Drop Target

Definable Drop Target

Definable Drop Target

Definable Drop Target

Definable Drop Target

Definable Drop Target

Definable Drop Target

1.

2.

3.

4.

5.

6.

7.

Reset Fluids

Osmotic Fluid Shifts

Clearly an EQUI-osmolar rehydration solution would result in no net shifts either in or out…

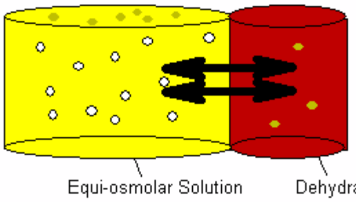

Osmotic Fluid Shifts

Clearly an EQUI-osmolar rehydration solution would result in no net shifts either in or out.

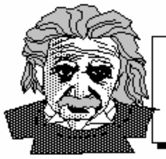

...but, what is less obvious, is that an equi-osmolar solution takes maximal advantage of **facilitated** mechanisms.

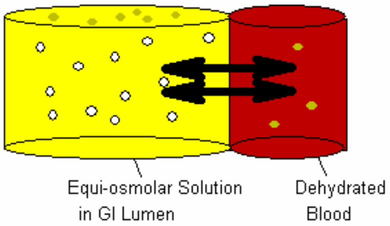

Section 4

Active Absorptive Processes

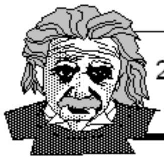

2. ORS takes best advantage of the active absorptive processes that remain intact.

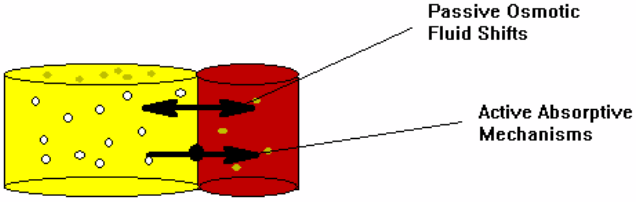

Active Absorptive Processes

The GI brush border is responsible for nutrient and fluid absorption.

Under normal circumstances, villus cells are responsible for absorption while crypt cells are responsible for secretion.

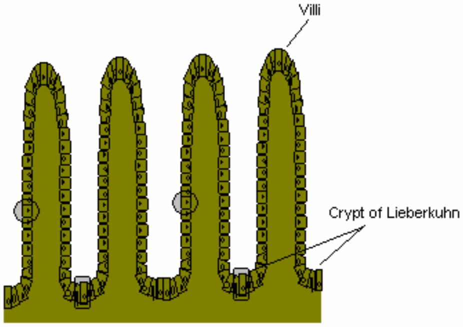

Active Absorptive Processes

The GI brush border is responsible for nutrient and fluid absorption. Under normal circumstances, villus cells are responsible for absorption while crypt cells are responsible for secretion.

In Enteroinvasive Diarrhea, viral pathogens and cytopathic bacteria destroy villus tip cells yet leave secretion cells unimpaired.

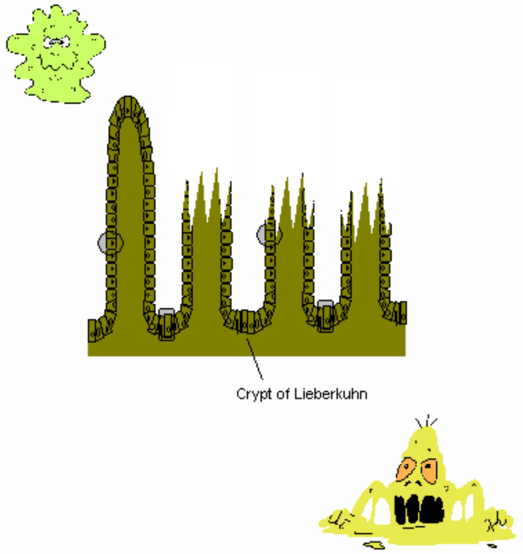

Active Absorptive Processes

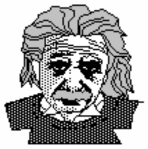

Acute gastroenteritis rarely interferes with the co-transporter pathways that bring in glucose or galactose with sodium.

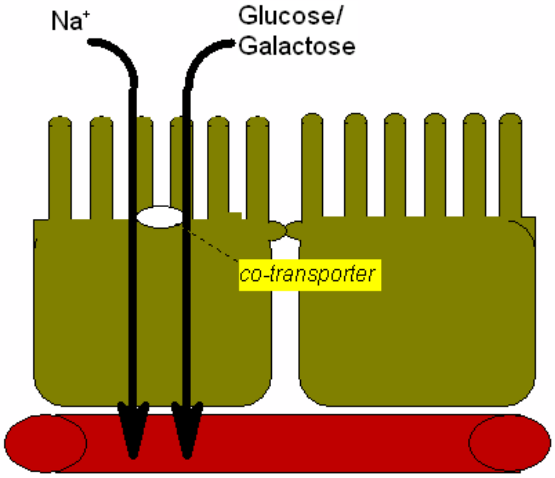

Active Absorptive Processes

The key here is that this Na+/glucose facilitated diffusion remains active in the crypt cells.

This allows these normally secretory cells to absorb.

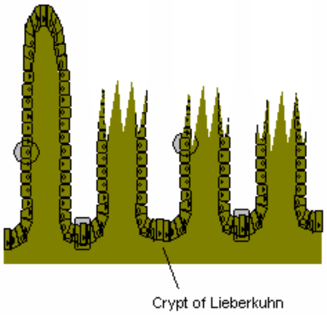

Active Absorptive Processes

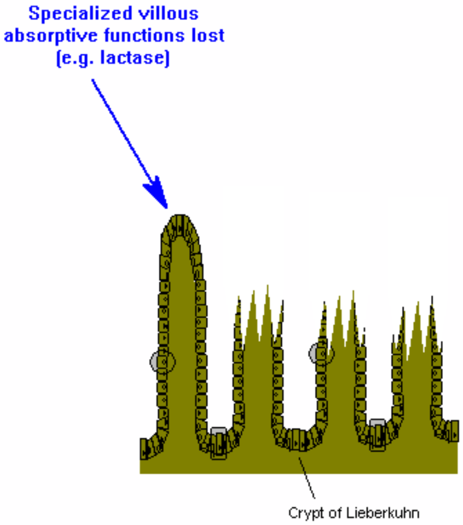

The key here is that this Na+/glucose facilitated diffusion remains active in the crypt cells.

This allows these normally secretory cells to absorb.

Active Absorptive Processes

The key here is that this Na+/glucose facilitated diffusion remains active in the crypt cells.

This allows these normally secretory cells to absorb.

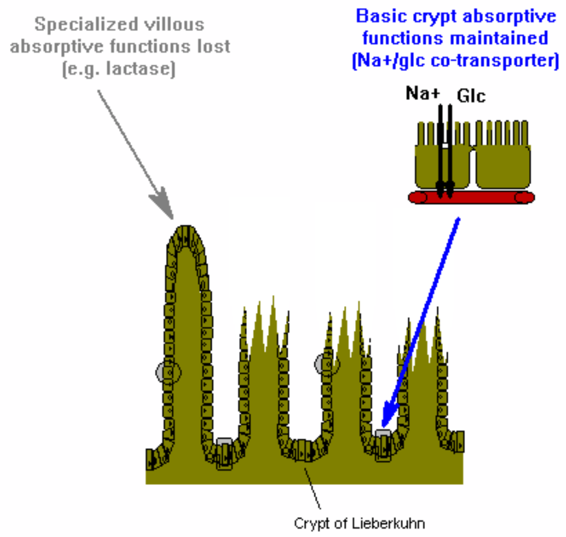

Section 5

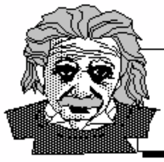

Let's see how ORS takes best advantage of the active absorptive processes that remain intact.

Basic crypt absorptive functions maintained (Na+/glc co-transporter)

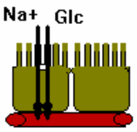

Example - Two Solutions

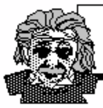

Consider the following two solutions:

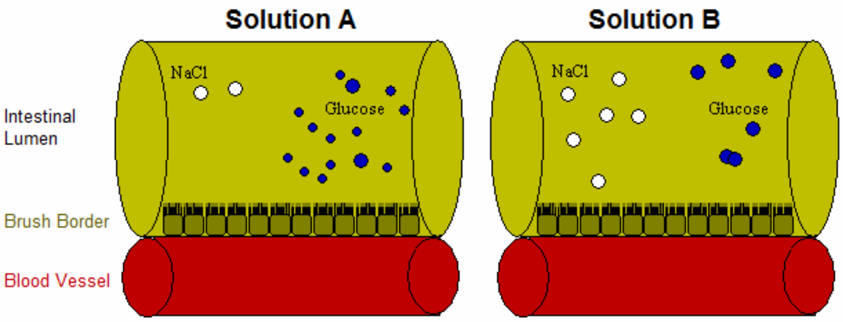

Example - Two Solutions

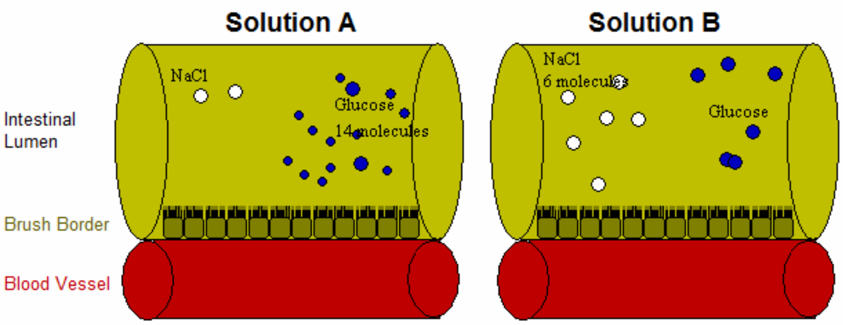

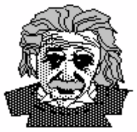

Which solution has the higher osmolarity?

- Solution A
- Solution B
- Both have equal osmolarity

Example - Two Solutions

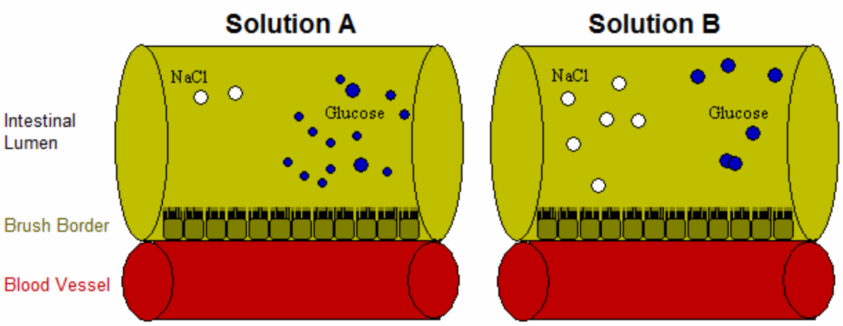

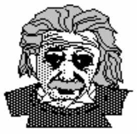

Which is the more effective Oral Rehydration Solution?

- Solution A
- Solution B
- Both are equally effective

Example - Two Solutions

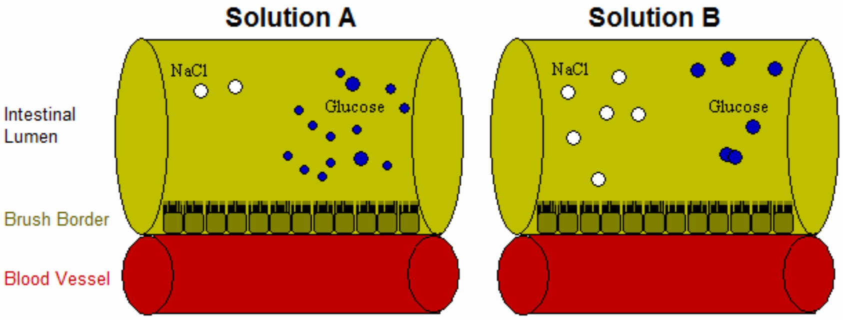

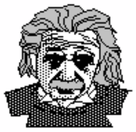

The ideal ratio of glucose molecules to sodium is 1:1
This ratio takes maximum advantage of Na/Glucose co-transport.

Example - Two Solutions

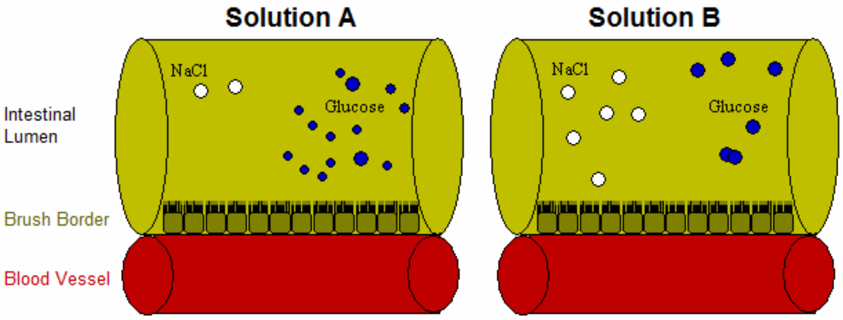

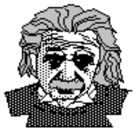

Think of it as a country hoe-down where only the paired ones get to go dance !!!!

Example - Two Solutions

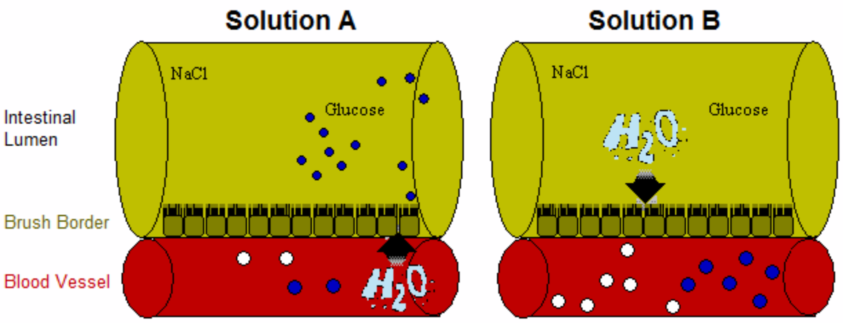

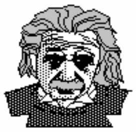

Notice the osmotic gradient now -- Solution B has a favourable gradient for the absorption of water whereas Solution A's would still aggravate a secretory diarrhea.

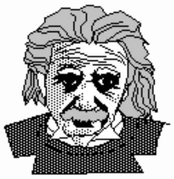

Let's relate this theory to some real-life fluids.

Real-life Fluids

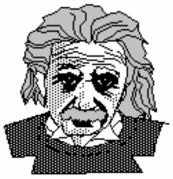

What do you notice on this table?

Solution	Glucose (mmol/L)	Na (mEq/L)	Ratio Glc : Na	Osmolarity (mOs/L)
Pedialyte	140	45	3	250
WHO	140	75	2	310
Gatorade	255	20	13	330
Cola	700	2	350	750
Apple Juice	690	3	230	730

Real-life Fluids

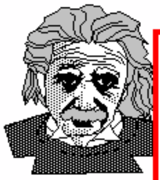

Note that the Na:Glucose ratios of the so-called "non-physiologic" liquids are astronomically higher than those for ORS solutions.

Gatorade is not great but not bad.

Solution	Glucose (mmol/L)	Na (mEq/L)	Ratio Glc : Na	
Pedialyte	140	45	3	
WHO	140	75	2	
Gatorade	255	20	13	[illegible]0
Cola	700	2	350	750
Apple Juice	690	3	230	730

Real-life Fluids

Check the osmolarities of the fluids.

High osmolarities inhibit absorption and promote secretory diarrhea.

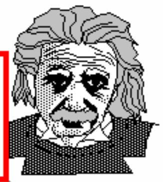

Solution	Glucose (mmol/L)	Na (mEq/L)	Ratio Glc : Na	Osmolarity (mOs/L)
Pedialyte	140	45	3	250
WHO	140	75	2	310
Gatorade	255	20	13	330
Cola	700	2	350	750
Apple Juice	690	3	230	730

Summary

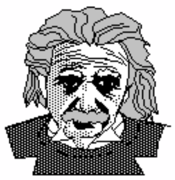

In summary, remember the 2 advantages that Oral Rehydration Solutions have over other available fluids:

1. ORS causes less in the way of deleterious osmotic fluid shifts.

2. ORS takes best advantage of the active absorptive processes that remain intact.
