# Supplementary material for: Reinforcing outpatient medical student learning using brief computer tutorials: the Patient-Teacher-Tutorial sequence
Source: BMC Med Educ. 2012 Aug 8;12:70. doi: 10.1186/1472-6920-12-70 (PMC3517358; doi:10.1186/1472-6920-12-70)
Supplement: Additional file 2 — Test questions. All test questions used in the study. [file 1472-6920-12-70-S2.pdf]

Just-in-Time Learning
Pediatric Emergency Medicine

# POST-TEST

This sheet will be detached at the time of marking the examination.

1. Please write into the box the code word that you had come up with at the time of the pre-test.

| |
|---|
| |

2. Write the same word on the yellow post-it and hand it to the preceptor.

3. What is today's date? ________________________

4. Which computer tutorials did you do?

   - ☐ Fever without source (FWS) Only
   - ☐ Oral Rehydration (ORS) Only
   - ☐ FWS and ORS (Both)
   - ☐ Neither one

5. When did you do your tutorials?

- ☐ I did my tutorial immediately after reviewing the patient with your preceptor.
- ☐ I saw at least one other patient before doing the tutorial.
- ☐ I did the tutorial the next day or later after seeing the index case.

6. Do you prefer doing similar tutorials:

- ☐ Immediately after reviewing the patient with your preceptor?
- ☐ Later that day?
- ☐ Later that week for review?
- ☐ No preference?

If you had a preference, can you tell us why?

7. Any other comments on the computer tutorials and their integration into a medical student’s emergency medicine experience?

*Instructions: You have 15 minutes to complete this examination. There are 16 questions totaling 55 points.*

1. A 17-day old infant has a fever. List the 3 organisms that can cause bacteremia/sepsis in this age group AND that drive our antibiotic choices. (*3 points*)

2. Which two bacteria are most likely to cause bacteremia/septicemia in a 9-month old febrile infant? (*2 points*)

3.  List 5 clinical factors (history and physical) that increase the risk of bacteremia/sepsis.
*(5 points)*

4.  A 39-day old infant presents to the ED with a 1-day history of fever to 101F (38.3C).  What are the laboratory parameters (Philadelphia Criteria) that predict low risk of serious sequelae?  *(5 points)*

5.  A well appearing 39-day old infant is febrile.  There is no significant past medical history.  We can largely decide on whether to admit a 39-day old febrile infant based on laboratory criteria.

□ True or □ False?  *(Circle One -  2 points)*

6. What is meant by the term "Fever Without Source" for children presenting to the ED. Why is this concept important? *(5 points)*

7. Choose one laboratory test (or not) from the right hand side for each of the scenarios below:

Except where specified, all children in the scenarios have been vaccinated, are well appearing and have appropriate followup. *(1 point each)*

QUESTION DROPPED

| **Circle One Response** | **Cas[illegible] Des[illegible]n** | | **Possible Responses** |
|---|---|---|---|
| 1 2 3 4 *(Circle One)* | A 9 month old female with fever up to 103[illegible] (39.4) for 4 days. | | 1. CBC<br>2. CXR<br>3. Urine Culture<br><br>4. No tests at this time |
| 1 2 3 4 *(Circle One)* | A 6 month old with fever to 104F (40C) & rhinitis for 2days. | | |
| 1 2 3 4 *(Circle One)* | An unimmunized 13 month old male presents with fever to 101 (38.5C) for 1 day | | |
| | | | |

**8.  A 25-day old infant presents to you having had a fever during the day. Apart from mild rhinitis, the child has otherwise been asymptomatic. The rest of the family all have cold symptoms.  Physical examination reveals a perfectly-well child save for a Temperature of 38.2C (100.8F) taken rectally.**

**Which one of the following would you do?** *(2 points)*

- ☐ Full Septic Workup and Admit Automatically
- ☐ Full Septic Workup and Admit if Positive
- ☐ Partial Septic Workup – Further Workup if Positive
- ☐ No workup necessary

**9.  A 6-week old infant presents to you with the same scenario as the previous child:  mild rhinitis; otherwise asymptomatic; the rest of the family all have cold symptoms.  Physical examination is normal except a tempterature of 38.2C (100.8)  taken rectally.**

**Which one of the following would you do?** *(2 points)*

- ☐ Full Septic Workup and Admit Automatically
- ☐ Full Septic Workup and Admit if Positive
- ☐ Partial Septic Workup – Further Workup if Positive
- ☐ No workup necessary

10. **A 3-month old child presents to you with the same scenario as the previous infant except the temperature is higher at 39.5C.  The WBC is measured and found to be 16,000.**

> **mild rhinitis; otherwise asymptomatic; the rest of the family all have cold symptoms; physical examination is normal.**

**What is the probability that this child will suffer permanent sequelae as a result of this illness?** *(2 points)*

- ☐ **1 in 500**
- ☐ **1 in 1000**
- ☐ **1 in 2000**
- ☐ **1 in 5000**

11. Compare the composition of Pedialyte with Apple Juice. What factors make Pedialyte a better choice than diluted apple juice for oral rehydration? *(5 points)*

12. What is the mechanism of diarrhea in gastroenteritis as seen in our Emergency Department? Use as much detail in your answer as possible. *(5 points)*

13. How does the use of Oral Rehydration Solutions circumvent the pathophysiology you described above? *(5 points)*

14. Why is ORS better than other fluids for children with diarrhea? Check ALL that are correct. *(3 points)*

- ☐ ORS is less osmotically active
- ☐ ORS is more nutritious
- ☐ ORS takes advantage of passive diffusion
- ☐ ORS takes advantage of facilitated transport

15. List the osmolarity of the following fluids: *(4 points)*

| **Fluid** | **Osmolarity** |
|---|---|
| | |
| Pedialyte | |
| | |
| Apple Juice | |
| | |
| Gatorade | |
| | |
| Coca-Cola | |
| | |
| Serum Osmolarity | 290 mOsm/Liter |
| | |

16. Co[illegible]ared with Pedialyte, Gatorade has: *(2 poi[illegible])*

QUESTION DROPPED

- ☐ More glucose, M[illegible]re s[illegible]d[illegible] [illegible]hl[illegible]ide
- ☐ More glucose, Equal sodi[illegible] ch[illegible]
- ☐ More glucose, Less sodium chloride
- ☐ Less glucose, Less sodium chloride
